# Selection of housekeeping genes for gene expression studies in the adult rat submandibular gland under normal, inflamed, atrophic and regenerative states

**DOI:** 10.1186/1471-2199-9-64

**Published:** 2008-07-17

**Authors:** Nicholas Silver, Emanuele Cotroneo, Gordon Proctor, Samira Osailan, Katherine L Paterson, Guy H Carpenter

**Affiliations:** 1 Salivary Research Unit, Floor 17, Guy's Tower, King's College London, London, UK

## Abstract

**Background:**

Real-time PCR is a reliable tool with which to measure mRNA transcripts, and provides valuable information on gene expression profiles. Endogenous controls such as housekeeping genes are used to normalise mRNA levels between samples for sensitive comparisons of mRNA transcription. Selection of the most stable control gene(s) is therefore critical for the reliable interpretation of gene expression data. For the purpose of this study, 7 commonly used housekeeping genes were investigated in salivary submandibular glands under normal, inflamed, atrophic and regenerative states.

**Results:**

The program NormFinder identified the suitability of *HPRT *to use as a single gene for normalisation within the normal, inflamed and regenerative states, and *GAPDH *in the atrophic state. For normalisation to multiple housekeeping genes, for each individual state, the optimal number of housekeeping genes as given by geNorm was: *ACTB*/*UBC *in the normal, *ACTB*/*YWHAZ *in the inflamed, *ACTB*/*HPRT *in the atrophic and *ACTB*/*GAPDH *in the regenerative state. The most stable housekeeping gene identified between states (compared to normal) was *UBC*. However, *ACTB*, identified as one of the most stably expressed genes within states, was found to be one of the most variable between states. Furthermore we demonstrated that normalising between states to *ACTB*, rather than *UBC*, introduced an approximately 3 fold magnitude of error.

**Conclusion:**

Using NormFinder, our studies demonstrated the suitability of *HPRT *to use as a single gene for normalisation within the normal, inflamed and regenerative groups and *GAPDH *in the atrophic group. However, if normalising to multiple housekeeping genes, we recommend normalising to those identified by geNorm. For normalisation across the physiological states, we recommend the use of *UBC*.

## Background

Saliva is secreted by three pairs of major salivary glands – parotid, submandibular and sublingual – as well as numerous other minor salivary glands located around the mouth. Understanding atrophy and regeneration of salivary glands is clinically important, and studies have shown that acinar cells, which are lost from atrophic salivary glands, re-differentiate from remaining duct cells [1-3]. Studies have also shown that mitotic proliferation of remaining acinar cells participates in the increase of acini in the regenerative process of parotid glands where acinar cells remain [4,5], and newly differentiated acinar cells proliferate actively in the regeneration of atrophic parotid glands in the absence of residual acinar cells [6]. In the submandibular gland, which differs histologically from the parotid gland, proliferation during regeneration has also been demonstrated [7]. Furthermore, recent observations of embryonic like ductal branch structures in regenerating submandibular tissue has led to a preliminary link between regeneration and embryonic development [8]. Which key gene expression signals induce apoptosis in atrophy and subsequent regeneration of the salivary gland still remains unclear. In order to help answer these questions, quantitative real time reverse transcription polymerase chain reaction (Q-RT-PCR) has been employed to study gene expression in the rat salivary gland model.

When comparing gene expression in different samples, it is essential to consider experimental variations such as amount of starting material, RNA extraction and reverse transcription efficiencies. To account for these, accuracy of Q-RT-PCR relies on normalisation to an internal control, often referred to as a reference or housekeeping gene. The prerequisite of a suitable housekeeping gene is that it should, of course, be adequately expressed in the tissue of interest, and that it shows minimal variability in expression between samples and under different experimental conditions used [9,10].

Commonly used housekeeping genes in Q-RT-PCR include beta actin (*ACTB*), glyceraldeyde-3-phosphate dehydrogenase (*GAPDH*), ribosome small subunit (18S) ribsosomal RNA (rRNA), Ubiquitin C (*UBC*), hypoxanthine guanine phosphoribosyl transferase (*HPRT*), succinate dehydrogenase complex, subunit A (*SDHA*) and Tyrosine 3-monooxygenase/tryptophan 5-monooxygenase activation protein, zeta polypeptide (YWHAZ) [11-13]. However, many of these control genes can show unacceptable variability in expression [14-19]. An example of this was shown by Torres et al [20], where mRNA levels of *GAPDH *were found to be androgen-dependent and varied according to the experimental conditions. Furthermore, Suzuki et al [21] discussed the uses and pitfalls of using 'classical' control genes such as *GAPDH *and ACTB. Here, cases were highlighted in which these control genes had been shown to be significantly modulated, and as a result, precautionary measures were suggested, such as the use of more than one control gene for normalisation. Without appropriate normalisation, expression profiles of target genes will likely be misinterpreted [14-18]. With increased gene expression profiling in pre-clinical genetic research, in parallel with ambitions for improved oral health, a need for accurate control genes for the various physiological states of the rat submandibular gland has emerged; however, appropriate studies in this area have not yet been conducted.

In this study, we chose to investigate a panel of 7 housekeeping genes (Table 1) in the adult rat submandibular gland in normal, inflamed, atrophic and regenerative states.

**Table 1:** Panel of 7 candidate housekeeping genes selected

**Gene symbol**	**Gene Name**	**mRNA accession number**	**Function**
*ACTB*	Beta-actin	NM_031144	Cytoskeletal structural protein
*ARBP*	Acidic ribosomal phosphoprotein P0	NM_022402	Ribosomal structural protein/nucleic acid binding
*GAPDH*	Glyceraldeyde-3-phosphate dehydrogenase	NM_017008	Glycolytic enzyme
*HPRT*	Hypoxanthine guanine phosphoribosyl transferase	NM_012583	Involved in the metabolic salvage of purines in mammals
*SDHA*	Succinate dehydrogenase complex, subunit A, flavoprotein	NM_130428	Involved in the oxidation of succinate
*UBC*	Ubiquitin C	NM_017314	Possible involvement in protein catabolism
*YWHAZ*	Tyrosine 3-monooxygenase/tryptophan 5-monooxygenase activation protein, zeta polypeptide	NM_013011	Protein domain specific binding

These different states were induced using intra-oral duct ligation, a well characterised surgical technique [22,23], whereby the submandibular duct is clipped (ligated) to induce initially inflammation, after 2 wks extensive atrophy, and subsequently removed (de-ligated) to induce regeneration [8,22-24]. Specifically, the states were defined as: unoperated (normal), 24 hr ligated (inflamed), 2 wk ligated (atrophic), and 2 wk ligated glands followed by 3 days of de-ligation (regenerative). Furthermore, we evaluated a selected number of housekeeping genes via normalisation of a target gene, here *SMGB *[25,26], a gene which encodes a secretory protein identified in the development and regeneration of the submandibular gland [8].

## Results

### Gland Weights

**Figure 1:**
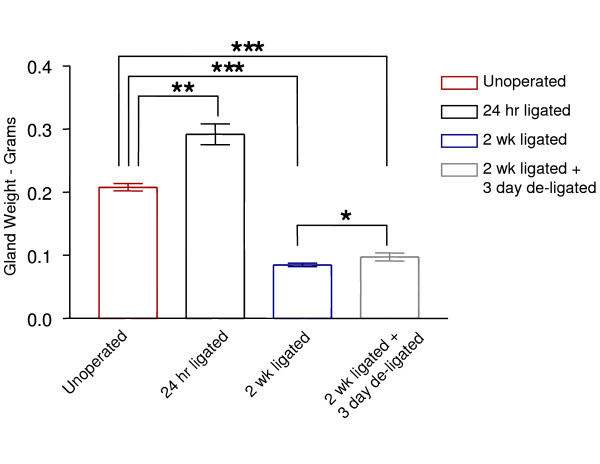
**Mean weight ± SEM of unoperated, 24 hr ligated, 2 wk ligated and 2 wk ligated + 3 day de-ligated submandibular glands**. * indicates statistical significance P < 0.05, ** P < 0.01 and *** P < 0.001 and were calculated using the Student's t-test (n = 5).

In order to provide evidence that the surgical procedures carried out in the ligation/de-ligation model were successful, the weight of the rat submandibular glands were measured and recorded. Following 24 hrs of ligation, submandibular glands showed an increased weight of approximately 40% (P < 0.01) when compared to the unoperated glands (Figure 1). Following 2 wks of ligation, submandibular glands were less than 50% (P < 0.001) of the unoperated controls. However, 2 wk ligated + 3 day de-ligated glands showed a small but significant increase in weight (P < 0.05) when compared with the glands ligated for 2 wks.

### Histology

**Figure 2:**
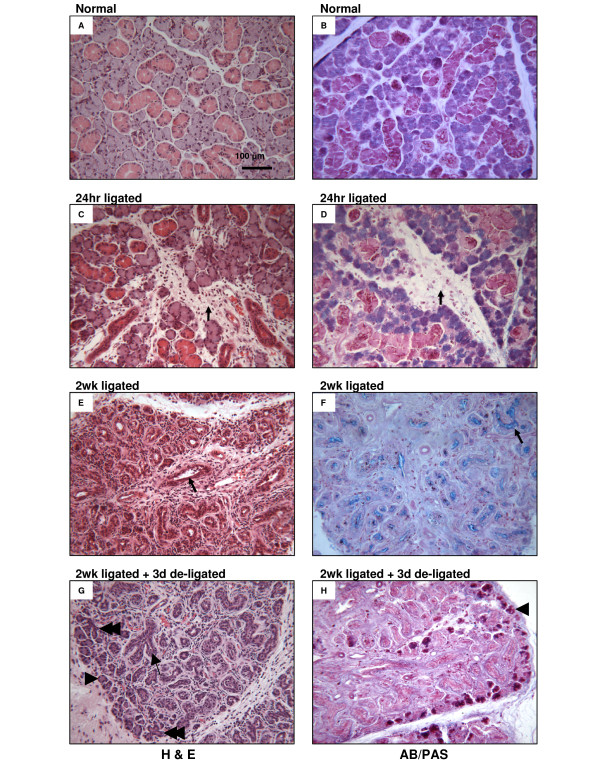
**A-H: H & E and AB/PAS staining**. (A & B) Control: Normal unoperated submandibular gland showing the typical appearance of acini and ductal cells. (C & D) 24 hr ligated: Large numbers of infiltrating inflammatory cells in the increased stroma and interstitial spaces (arrows). (E) 2 wk ligated – H & E: Luminal dilation of ducts (arrow), absence of acini, and extensive inflammation. (F) 2 wk ligated – AB/PAS: Loss of cellular secretory granules and presence of material in the lumen of ducts (arrow). (G) 3 days following removal of 2 wk ligation – H & E: Some acini (arrow head) and ductal cells (arrow) have recovered some of their size, while acinar-ductal branch structures are also visible (double arrow head). (H) 3 days following removal of 2 wk ligation – AB/PAS: Some acini have recovered their glycoprotein content (arrow head).

When compared with the unoperated normal glands, the increased weight of the 24 hr ligated glands was accompanied by changes in the histology as illustrated by haematoxylin-and-eosin (H&E) and alcian blue-periodic acid-Schiff (AB/PAS) staining (Figure 2a–d). Compared with the unoperated submandibular gland, the 24 hr ligated gland appeared less tightly packed and contained large numbers of infiltrating inflammatory cells. These cells were most prominent in the stroma of the gland although they were also present within the interstitial space between acini and ductal units. The infiltrating cells appeared to be mostly neutrophils and macrophages (based on cell size and shape) although no detailed analysis was attempted. In addition, blood vessels and salivary striated duct lumena appeared dilated in the ligated gland compared with the normal gland. In the 2 wk ligated glands, when compared with the unoperated glands, they showed increased inflammatory cell infiltration in the connective tissue between the lobules and among the parenchymal elements (Figure 2e–f). H&E staining of the 2 wk ligated glands suggested the acini had almost completely disappeared, whilst many residual duct like structures were still visible with considerably dilated lumens. In addition, AB/PAS showed an almost complete absence of secretory granules from both acini and ductal cells in the 2 wk ligated glands. The stain also showed the presence of some secretory products in the lumen of the ducts (Figure 2f) in contrast to the de-ligated glands where the AB/PAS staining in the lumen was lost (Figure 2h).

In a similar fashion to the 2 wk ligated, the 3 day de-ligated glands, when compared with the unoperated, showed increased inflammatory cell infiltration in the connective tissue between the lobules and among the parenchymal elements (Figure 2g–h). The H&E staining showed that some acini, mostly on the edge of the lobules had recovered some of their size and the lumen of the ducts appeared less obvious. In addition, embryonic type developmental branch structures were apparent as indicated (Figure 2g – double arrow heads). Further evidence from the AB/PAS staining showed that several acini, mainly located at the edges of the lobules had recovered some of their glycoprotein content.

### RNA Quality

RNA purity was measured using the NanoDrop^® ^Spectrophotometer (NanoDrop Technologies). The mean (± SEM) A_260/280 _ratio of RNA samples was 2.07 ± 0.04 (range from 1.96–2.12) and reflected pure and protein free RNA. The mean (± SEM) A_260/230 _ratio was 1.98 ± 0.21 (range from 1.63 – 2.23) and indicated the RNA was phenol and ethanol free. The RNA integrity as an essential quality criterion was characterised by the RNA integrity number (RIN) [27], measured on the Agilent 2100 Bioanalyzer (Agilent Technologies). The mean (± SEM) RIN value of all RNA samples was 8.7 ± 0.53 (range from 7.8–9.5).

### Q-RT-PCR efficiency and intra- and inter- assay variability

Quantitative real-time PCR was used to measure the RNA transcription level of a number of candidate housekeeping genes (Table 1). To compare different RNA transcription levels, the Ct values were compared directly. The Ct is defined as the number of cycles needed for the fluorescence to reach a specific threshold level of detection and is inversely correlated with the amount of template nucleic acid present in the reaction [28]. To ensure optimal comparability between the PCR assays, efficiency of each individual assay was determined by measuring serial dilutions of cDNA from a control (unoperated) submandibular gland in duplicate. Only Ct values <40 were used for calculation of the PCR efficiency from the given slope generated in the 7500 system software v1.3.1 according to the equation: PCR efficiency = (10^[-1/slope]^-1) × 100. All PCR assays displayed efficiencies between 90 and 102% (Table 2). Intra and inter-assay variation was investigated in three independent runs performed on three consecutive days. Intra-assay variation was <0.4% and inter-assay variation <1.0%.

**Table 2:** Characteristics of primer and probe sets

**Gene symbol**	**Context Sequence**	**Amplicon length**	**Efficiency (%)**	**Primer & Probe Set**
*ACTB*	CTTCCTGGGTATGGAATCCTGTGGC	91	100	Assay by design (ABI)
*ARBP*	TGGCCAATAAGGTGCCAGCTGCTGC	100	90	Assay by design (ABI)
*GAPDH*	CGGGAAACCCATCACCATCTTCCAG	87	102	Assay by design (ABI)
*HPRT*	ACTGGAAAGAACGTCTTGATTGTTG	100	94	Assay by design (ABI)
*SDHA*	CATACTGTTGCAGCACAGGGAGGTA	59	98	Assay by design (ABI)
*UBC*	TGGGTTTGATGGGGAGGTGTCTTAG	88	98	Assay by design (ABI)
*YWHAZ*	GCAACGACGTACTGTCTCTTTTGGA	104	102	Assay by design (ABI)

### Expression profiling of housekeeping genes

**Figure 3:**
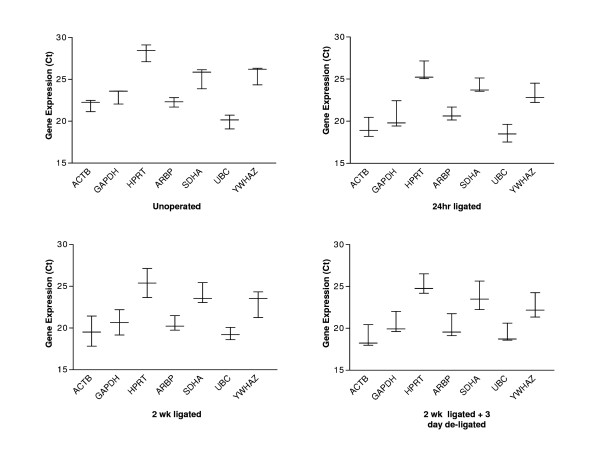
**Q-RT-PCR cycle threshold (Ct) values for 7 candidate housekeeping genes among 20 rat submandibular samples**. Gene expression levels are shown as medians (lines) and ranges (whiskers). Candidate housekeeping genes include *ACTB*, *GAPDH*, *HPRT*, *ARBP*, *SDHA*, *UBC *and *YWHAZ*.

Primers were selected for commonly used housekeeping genes for a total of 7 control genes – see Table 2 for details. Particular attention was paid to selecting genes that belong to different functional classes, which significantly reduces the chance that genes might be co-regulated. The expression level of the 7 internal control genes was determined in 20 submandibular gland samples, comprising of 4 different groups – 5(unoperated samples), 5(24 hr ligated), 5(2 wk ligated) and 5(2 wk ligated + 3 day de-ligated) (Figure 3).

### Stability of housekeeping genes within sample groups

Stability of the 7 housekeeping genes was first assessed for each sample group. Gene expression levels were measured by real-time PCR and the expression stabilities were evaluated by the two most commonly used software based methods: (1) geNorm [29] and (2) NormFinder [30]. GeNorm uses a gene-stability measure M, which is defined as the average pairwise variation between a particular gene and all other control genes and calculates the optimal number of genes necessary for normalisation of a target gene. The NormFinder program uses a model-based approach for estimation of expression and enables the identification of the single best gene as well as giving a ranking order. Based on the M value, all housekeeping genes in all but one group (the 2 wk ligated) reached the geNorm arbitrary cut-off level of 0.5 for stability (Table 3). This suggests that the use of any of these housekeeping genes for normalisation is warranted. For the 2 wk ligated group, those genes which were within the stable 0.5 cut-off limit were *ACTB*, *HPRT *and *GAPDH *(Table 3). The optimal number of housekeepers for normalisation as given by geNorm can be seen in Table 4. These were as follows: *ACTB*/*UBC *in the unoperated control group, – *ACTB*/*YWHAZ *for the 24 hr ligated, – *ACTB*/*HPRT *for the 2 wk ligated group and – *ACTB/GAPDH *for the 2 wk ligated + 3 day de-ligated group. NormFinder identified *HPRT *as the single most stable gene for the unoperated and 24 hr ligated groups, *GAPDH *for the 2 wk ligated, and *HPRT *for the 2 wk ligated + 3 day de-ligated group (Table 4).

**Table 3:** Housekeeping gene rankings for each tissue state

**Housekeeping genes ranked in order of their expression stability**							
**Unoperated**	**M**	**24 hr ligated**	**M**	**2 wk ligated**	**M**	**2 wk ligated + 3 day de-ligated**	**M**

*ARBP*	0.434	*SDHA*	0.496	*SDHA*	0.792	*SDHA*	0.387
*SDHA*	0.364	*GAPDH*	0.417	*YWHAZ*	0.641	*YWHAZ*	0.318
*YWHAZ*	0.328	ARBP	0.352	*UBC*	0.562	ARBP	0.272
*GAPDH*	0.243	*UBC*	0.278	ARBP	0.505	*HPRT*	0.207
*HPRT*	0.196	*HPRT*	0.213	*GAPDH*	0.359	*UBC*	0.179

*ACTB*-*UBC*	0.155	*ACTB*-*YWHAZ*	0.136	*ACTB*-*HPRT*	0.226	*ACTB*-*GAPDH*	0.124

Increasing stability from top to bottom; the two most stable control genes in tissue state, for example *ACTB *and *UBC *in unoperated tissue, cannot be ranked in order because of the required use of gene ratios for gene stability measurements. M is defined as the control gene-stability measure.

**Table 4:** Summary and comparison of the top candidate housekeeping genes for each gland state identified by geNorm and NormFinder

	**geNorm**	**NormFinder**
Unoperated	*UBC/ACTB*	*HPRT*
24 hr ligated	*YWHAZ/ACTB*	*HPRT*
2 wk ligated	*HPRT/ACTB*	*GAPDH*
2 wk ligated + 3 day de-ligated	*GAPDH/ACTB*	*HPRT*

For the geNorm program, determination of the optimal number of control genes for normalisation is made. This is calculated through pairwise variation analysis through which Vandesompele et al proposed 0.15 as a cut off value. This optimal number of housekeeping genes is in the order given by the program.

### Stability of housekeeping genes compared to normal tissue

**Figure 4:**
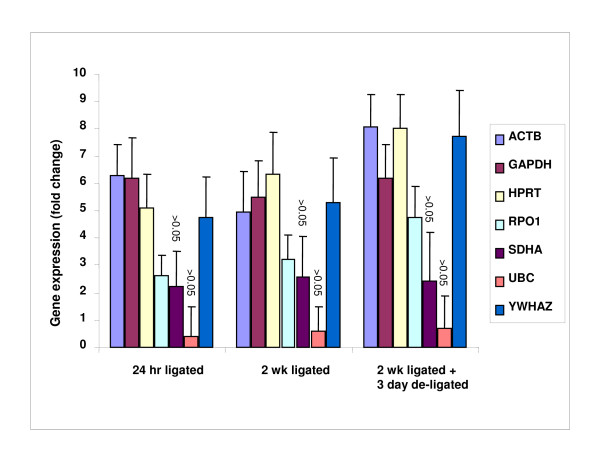
**Candidate housekeeping gene expression normalised to total RNA and relative to the normal state**. In the figure n = 5 per experimental group and error bar represent ± SD. The Mann Whitney-U test was used to test for differences between sample sets. *UBC *and *SDHA *were the only two genes which showed no significant difference (>0.05) throughout the different groups when compared with the control group.

**Figure 5:**
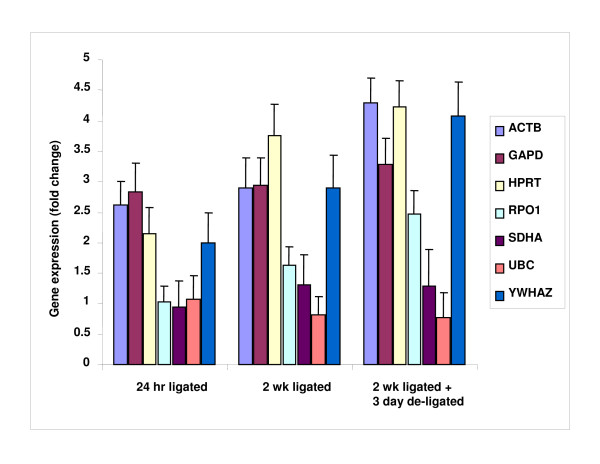
**Candidate housekeeping gene expression following geometric normalisation and relative to the normal state**. Candidate housekeeping genes were normalised by dividing the relative expression value by the geometric mean of the expression levels of the two selected housekeeping genes *UBC *and *SDHA*.

Stability of the 7 housekeeping genes was further assessed in the 24 hr ligated, 2 wk ligated and 2 wk ligated + 3 day de-ligated groups in comparison to the unoperated state. Expression stability was evaluated by (1) RNA normalised gene expression (2) The Mann-Whitney-U Test and Equivalence statistics (3) Normalisation to the geometric mean of selected housekeeping genes (4) NormFinder. Following normalisation to total RNA, gene expression levels were expressed relative to the unoperated group (Figure 4). The top 3 most stable housekeeping genes were: *UBC*, *SDHA *and *ARBP *for all states. Following statistical analysis using the Mann-Whitney-U Test, only *UBC *and *SDHA *for the 24 hr ligated, 2 wk ligated and 2 wk ligated + 3 day de-ligated states showed no change from the unoperated control group (>0.05) (Figure 4). The equivalence assessment was then carried out to look for similarities between the groups. In the present case, the variables that showed statistically significant differences indicated an approximately 10% change. Thus, equivalence cannot be assumed for the other variables where differences might be outside this level. Applied to UBC and SDHA, the variables for which equivalence testing was required, the upper limit of the 95% confidence intervals for UBC did not reach the 10% level of reduction for the unoperated vs 2 wk ligated & 2 wk ligated + 3 day de-ligated groups, implying equivalence. This was also true of SDHA for comparison of the unoperated vs 24 hr ligated group. However, the other comparisons all exceeded a 10% reduction and could not be regarded as equivalent. Using these methods as an indicator of housekeeping gene inter-group stability, we then normalised the data against the geometric mean of the expression levels of *UBC *and *SDHA*. A comparison of the geometric normalised expression of each of the housekeeping genes further demonstrated the stability of *UBC *and *SDHA *relative to the other genes within the relevant groups (Figure 5, Table 5). NormFinder has the option of defining groups within the samples and was used to further evaluate housekeeping gene inter-group stability. Based on the best combination of two housekeeping genes, the NormFinder program suggested normalising to *UBC *and *HPRT *when comparing the 24 hr ligated/2 wk ligated with the unoperated state, and *UBC *and *GAPDH *when comparing the 2 wk ligated + 3 day de-ligated and unoperated state – see Table 5.

**Table 5:** Summary and comparison of the top candidate housekeeping genes when compared to the unoperated state identified by geometric normalisation and NormFinder

	**Normalised (Geometric mean)**	**NormFinder**
Normal vs 24 hr ligated	*UBC, ARBP*	*UBC, HPRT*
Normal vs 2 wk ligated	*UBC, SDHA*	*UBC, HPRT*
Normal vs 2 wk ligated + 3 day de-ligated	*UBC, SDHA*	*UBC, GAPDH*

### Evaluation and validation of selected candidate housekeeping genes using SMGB

Following identification of the most stable housekeeping genes from the full panel, a method was needed for their evaluation. As described previously by our laboratory [8], a correlation between the state of the submandibular gland and expression of the protein Smgb has been shown. More specifically, following 2 wks of ligation, a decrease in Smgb protein levels were seen when compared with unoperated glands. However, following 3 days of de-ligation, although a decrease in Smgb protein levels were still seen when compared with normal glands, an increase was seen relative to the atrophic glands. Therefore, we expected these observations to be mirrored at the mRNA level.

**Figure 6:**
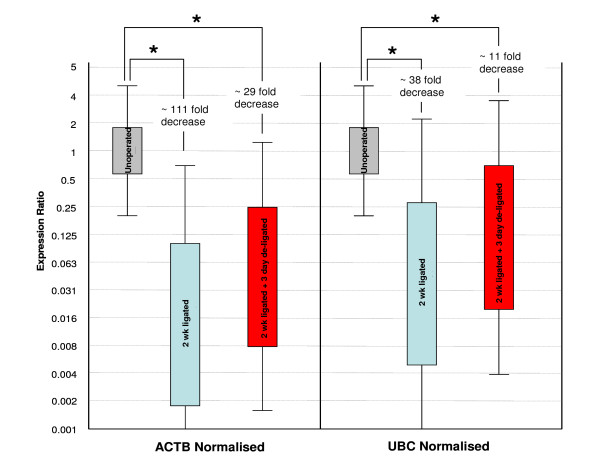
**Whisker box plots showing *SMGB *gene expression in terms of expression ratios**. The results are expressed as box plots of mRNA expression relative to the normal control state after normalisation to either ACTB or UBC. Shown are the 25% to 75% response ranges (top and bottom lines of boxes) and minima and maxima (whiskers). Asterisks indicate a significant change (p < 0.05) in SMGB expression in the 2 wk ligated and 2 wk ligated + 3 day de-ligated states when compared to the normal state. Changes in expression are also indicated in terms of fold change relative to the normal control level.

Consequently, gene expression analysis of *SMGB *in the 3 surgically induced groups was compared for normalisation using *ACTB*, one of the least stable housekeeping genes identified, and one of the highest scoring genes (most stable), *UBC*. This was carried out using the relative expression software tool (REST^©^) [31], developed for group-wise comparison and statistical analysis of relative expression results. When normalised to *ACTB*, *SMGB *was down-regulated in the 2 wk ligated sample group (in comparison to the normal unoperated group) by approximately 111 fold, P = 0.017 (Figure 6). However, in the 2 wk ligated + 3 day de-ligated group, although *SMGB *was down-regulated by approximately 29 fold, P = 0.038, an increase of approximately 3.8 fold was seen in comparison to the 2 wk ligated group (although this was not statistically significant, P = 0.388). When normalised to the more stable *UBC*, *SMGB *was down-regulated in the 2 wk ligated sample group (in comparison to the unoperated group) by approximately 38 fold, P = 0.016 (Figure 6). In the 2 wk ligated + 3 day de-ligated group, *SMGB *was down-regulated by approximately 11 fold, P = 0.044, however, an increase of approximately 3.6 fold was seen in comparison to the 2 wk ligated group (although again, this was not statistically significant, P = 0.388). Based on these results, evaluation of the two housekeeping genes for normalisation indicated that although the pattern of gene expression was identified as being similar to the protein expression using either *ACTB *or *UBC*, there was an approximately 3-fold under-estimation of target gene expression (*SMGB*) when normalising against *ACTB *when compared with *UBC*.

## Discussion

Characterisation of atrophy, followed by the early regeneration of the rat submandibular gland following intra-oral duct ligation [22,23] is crucial to fully understanding the genetic mechanisms leading to the recovery of various cellular elements, and ultimately their secretory ability. In addition, characterisation of these genetic mechanisms may not just be restricted to atrophy and regeneration of the rat submandibular gland, but may also have implications for the wider field of regenerative biology. In order to accurately detect and track these changes in gene expression level at the various stages of atrophy and regeneration, a sensitive and reproducible method is needed. Quantitative real-time PCR fulfils these requirements, in that it is one of the most established and sensitive methods available with which to detect gene transcript levels. As highly accurate quantitative gene expression data in Q-RT-PCR based gene expression studies are normalised relative to an internal control, it is critical to choose an appropriate control gene(s) for normalisation, so as not to misinterpret the expression profile of a target gene [14,17,32,33]. Since it is likely that no single control gene is stable for all studies [10], the selection of an appropriate control gene is necessary for each new experimental system, especially where complex tissues are involved.

To the best of our knowledge, there has not yet been a detailed evaluation of housekeeping genes in the rat submandibular gland. Moreover, there has not been a detailed study under different states of inflammation, atrophy and regeneration. In recent years, there have been a number of research papers and reviews evaluating the selection and effect of controls on normalised gene expression data in various rat tissues. One recent publication by Cai et al [34] involved the validation of housekeeping genes in a panel of rat tissues using low density arrays. Other studies in the rat model have focused on more specific tissues, including the retina [35], fetal brain [36], cortex and hippocampus [37] and liver [11]. However, none of these in depth housekeeping gene studies in the rat model have included the rat submandibular gland. Although no in depth studies are apparent in the submandibular gland tissue, there have been numerous research papers which have used single housekeeping genes to control for gene expression in this tissue. These have included the use of 18 S rRNA [38,39], *GAPDH *[40] and *ACTB *[41,42] for normalisation. Nevertheless, it is not always clear on what basis these various control genes were chosen, and of the various ones used, which were the most appropriate. As a consequence, in this study, we evaluated the gene expression stability of 7 commonly used 'classical' housekeeping genes in the rat submandibular gland, and furthermore, assessed the levels in states of inflammation (24 hr ligation), atrophy (2 wk ligation) and regeneration (2 wk ligation followed by 3 days of de-ligation).

In order to effectively assess the housekeeping gene expression levels, it was important to confirm the surgical procedures had been carried out successfully and this was done via inspection of both gland weight and histology. Ligation was confirmed in the 24 hr ligated glands, indicated by their heavier weight when compared with the normal glands (Figure 1). These heavier gland weights corresponded with the oedematous appearance, which was partially due to the build up of saliva, as well as due to inflammation related oedema seen in the histology as inflammatory infiltrate [24] (Figure 2c–d). In the 2 wk ligated glands, in addition to significant reduction in gland weight when compared to the normal unoperated glands, dramatic morphological changes occurred (Figure 2e–f), all of which suggested that the ligation of the excretory ducts was successful and consequently atrophy of the gland had ensued – this was as previously described in the literature [22,43-45]. After two weeks of ligation followed by 3 days of de-ligation, the weight of the de-ligated glands increased significantly over those undergoing just 2 wk ligation. In addition, acini cells, mostly on the edge of the gland lobules recovered their size (Figure 2g–h). Furthermore, absence of secretory material in the lumen of the gland ducts suggested reactivation of salivary flow [8]. Having confirmed all procedures, mRNA transcript levels of the different submandibular groups could be measured and compared with confidence.

Housekeeping gene expression stability 'within' sample groups was evaluated by (1) GeNorm applet and (2) NormFinder. The principle that the expression ratio of two ideal control genes should be identical across experimental groups is well established [29,46]. Based on this principle and using the geNorm program [29], we found that despite the dramatic morphological changes that had occurred in the submandibular tissue, the variability in housekeeping gene expression in each individual gland group was relatively low. This stability was reflected by the M values calculated for the 7 candidate housekeeping genes, which were all under the arbitrary threshold of 0.5 in the unoperated, 24 hr ligated and 2 wk ligated + 3 day de-ligated groups; except for the 2 wk ligated group in which only *ACTB*, *HPRT*, *GAPDH *were below this cut off value. The reasons for this are not clear but probably reflect the extremely different histology of an atrophic exocrine gland. As previously described, normalisation to a single housekeeping gene can lead to erroneous results [14,17,32,33]. Vandesompele et al [29] concluded that in order to measure gene expression levels accurately, normalisation by multiple housekeeping genes instead of one can be used. Consequently, a normalisation factor (NF) can be calculated, which is based on the geometric mean of a recommended minimum of housekeeping genes. Therefore, depending on practicality, we recommend using the geometric average of the two highest scoring housekeeping genes, as suggested by the geNorm program, here: *ACTB*/*UBC *in the unoperated group, – *ACTB*/*YWHAZ *for the 24 hr ligated, – *ACTB*/*HPRT *for the 2 wk ligated group and – *ACTB/GAPDH *for the 2 wk ligated + 3 day de-ligated group. Although expression stability analysis by NormFinder identified different top-ranking genes to geNorm (Table 4), the top ranking genes identified by NormFinder for each sample group were all identified by geNorm as being stable (according to the stability measure M). These were *HPRT *as the single most stable gene for the unoperated and 24 hr ligated groups, *GAPDH *for the 2 wk ligated, and *HPRT *for the 2 wk ligated + 3 day de-ligated group. This was also reported in a recent study by Lyng et al, whereby a different rank order between the geNorm and NormFinder programs was observed during identification of housekeeping genes in breast carcinomas. This difference in top-ranking between the two programs is likely reflective of the different embedded methods used. GeNorm uses a pairwise comparison approach of 'pairs of genes', whereas NormFinder uses a model-based approach for direct estimation of expression variation, as well as taking into account sample sub-groups. Based on these results, if normalising to multiple housekeeping genes, we suggest using those identified by geNorm. However, if normalising to a single housekeeping gene, we suggest using those identified by NormFinder, as they also agreed with those identified by geNorm as being stable.

Housekeeping gene expression stability 'between' sample groups was evaluated by (1) Normalising to total RNA (2) The Mann-Whitney-U Test and Equivalence statistics (3) Normalisation to the geometric mean of selected housekeeping genes (4) NormFinder. GeNorm was not used in this part of the analysis, as it does not provide the option of clearly defining sub-groups. However, NormFinder has an option to define sub-groups, making it more appropriate for comparison of the surgically induced states with the normal state. Following normalisation to total RNA, relative expression of the housekeeping genes in each group when compared with the normal unoperated group suggested the top 3 most stable genes to be *UBC*, *SDHA *and *ARBP*. Analysis was then carried out using the Mann-Whitney-U Test to look for statistically significant variation between the raw Ct values of the different groups. This analysis suggested that only the two genes *UBC *and *SDHA *showed no significant difference (>0.05) for the 24 hr ligated, 2 wk ligated and 2 wk ligated + 3 day de-ligated groups when compared with the unoperated group, therefore discounting the other genes in the panel as being sufficiently stable. Similarity between the groups was then assessed for UBC and SDHA by testing for equivalence. Here, equivalence was indicated for UBC in the unoperated vs 2 wk ligated and 2 wk ligated + 3 day de-ligated states. This was also true for SDHA for comparison between the unoperated and 2 wk ligated group. In situations where no optimal normalisation gene has been found, it may be prudent to normalise the data using the geometric mean based on multiple normalisation genes rather than a single gene [30]. The rationale for this is that the variation in the average of multiple genes is smaller than the variation in individual genes. Following this rationale, normalising the relative gene expression data to the geometric mean of *UBC *and *SDHA *further demonstrated the level of stability of these genes when compared with genes displaying the most variability, for example, *ACTB*, *GAPDH *and *HPRT*. In addition to using these more basic methods to detect stability, NormFinder was further incorporated into the analysis. Although NormFinder analysis of the sub-groups suggested *UBC *for use in normalisation, it also suggested using *HPRT *in combination. However, *HPRT *had previously been identified as being one of the more variable housekeeping genes between groups. This is likely explained by the fact that NormFinder uses a different approach to identifying stable genes. As mentioned previously, it uses a model-based approach for estimation of expression variation, uses definitions of sub-groups within a sample set, and has the build in ability to identify co-regulated genes based on the similarity of their expression profiles. It is possible therefore, that the NormFinder program has chosen the two genes, *UBC *and *HPRT*, on the basis that they show minimal co-expression. Nevertheless, having used multiple methods for stability analysis of the housekeeping gene panel between groups, *UBC *was consistently identified as being the most stable. We would therefore suggest the use of *UBC *when normalising between groups.

For the purpose of our gene expression studies in the submandibular gland, it is important that we are able to normalise as accurately as possible between the normal unoperated and surgically induced states. Therefore, in order to evaluate the selection of housekeeping genes chosen to normalise between tissue states, *SMGB *gene expression levels were normalised against *ACTB *and *UBC*, and compared with the observed protein levels previously measured by our group [8]. Here, patterns of *SMGB *mRNA expression were found to mirror that of their protein whether normalised to *ACTB *or *UBC*. However, and importantly, evaluation of the two housekeeping genes for normalisation in the surgically induced atrophic and regenerative states when compared to the normal state indicated that there was an approximately 3 fold under-estimation of target gene expression (*SMGB*) when normalising against *ACTB *when compared to the more stable *UBC*.

### Study Limitation

An important factor to consider regarding this study is the limited sample number (n = 5 for each group). Nevertheless, even with this low sample number and hence relatively low statistical power, results strongly suggested which genes were displaying the highest stability. This was likely aided by factors such as the high quality/integrity and purity of the RNA samples used, as well as the ability to accurately standardize the amount of RNA put into reactions. Furthermore, rats are relatively homogenous when compared with human samples, therefore, minimizing inter-sample variability.

## Conclusion

Induction of inflammation, atrophy and regeneration in the rat submandibular gland via intra-oral duct ligation serves as a useful model to study gene expression mechanisms. As a consequence, a survey of a small panel of the more commonly used housekeeping genes was carried out. Our study demonstrated the suitability of *HPRT *to use as a single gene for normalisation within the normal, inflamed and regenerative groups and *GAPDH *in the atrophic group. However, to further improve accuracy, we recommend normalising to multiple genes as suggested by the geNorm program in this study. These were: *ACTB*/*UBC *in the normal control state, *ACTB*/*YWHAZ *in the inflamed state, *ACTB*/*HPRT *in the atrophic state, and *ACTB*/*GAPDH *in the regenerative state. The most stable housekeeping gene identified between physiological states (compared to normal) was *UBC*, however, *ACTB*, which was identified as being one of the most stable genes within each state, was found to be one of the most variable.

## Methods

### Samples

Twenty adult male rats of Wistar (Harlan) strain were used and weighed 250–350 g at the time of experiment. All experimental and terminal procedures were conducted with approval of the local ethics committee and a Home Office license. Animals were divided into 4 groups: control (unoperated), short term ligated (inflamed), long term ligated (atrophic) and de-ligated (regenerative) group. The control gland group underwent no surgical procedure (5 rats), – the ligated group included glands ligated for either 24 hr (5 rats) or 2 wks (5 rats) using a micro-clip with a plastic tube (see below), – and for the de-ligated group, a separate set of glands ligated for 2 wks were then allowed to recover for a period of exactly 3 days (5 rats). Rats were killed by an overdose of pentobarbitone.

### Duct ligation

Animals were anaesthetised with Ketamine/Xylazine_I.P._(0.75 ml/kg of each drug giving a dose of 75 + 15 mg/kg_I.P., _respectively), given as a mixture of one part of each drug. The main excretory ducts of the right submandibular (RSM) and sublingual glands were carefully dissected with the help of a dissecting microscope through a small incision in the floor of the mouth from an intra-oral approach [22]. The ducts were ligated 5 mm posterior to the ductal orifice (without including the chorda lingual nerve) with a metal micro-clip (SLS, Vitalitec International, 35680 Domalain, France) and a plastic tube was inserted at the neck of the joint of the clip to avoid any damage and to minimize fibrosis of the ducts [23]. The incision in the floor of the mouth was closed with 8/0 Ethilon suture (Johnson & Johnson Intl, Brussels, Belgium). Animals were allowed to recover from anaesthesia in a cage maintained in a warm room. Aseptic conditions were used throughout the surgical procedure of duct ligation to reduce the risk of infection. Contralateral glands were not used as controls in this study because compensatory hyperplasia occurred when the opposite gland was extirpated.

### Duct de-ligation

Following ligation, under the same anaesthesia as mentioned earlier, a small incision in the floor of the mouth was made and a careful dissection around the micro-clip was performed without causing any damage to the duct. The clip was held with dissecting forceps and opened with a scalpel (no. 11) blade and then both micro-clip and plastic tube were gently pulled out and the incision closed with 8/0 suture. Animals were allowed to recover from anaesthesia in a warm room. In the case of this study de-ligation was always carried out following 2 weeks of ligation.

### Histochemical staining of tissue samples

At the end of experiments submandibular glands were removed under terminal anaesthesia, weighed and tissue sections fixed in formal sucrose over night. The rest of the gland was frozen at -80°C for further processing and analysis. Tissue sections were then dehydrated in a series of alcohols, embedded in paraffin wax and 10 μm sections stained with Ehrlich Haematoxylin and 1% Eosin (H & E) for general morphology. For the demonstration of acinar cells and secretory granules Alcian Blue and periodic acid Schiff's stains (AB/PAS) were used [47].

### Isolation of RNA and reverse transcription

Total RNA was isolated from approximately 30 mg of liquid nitrogen preserved submandibular gland tissue and homogenized in 600 μl RNA-Bee™ using a 7 ml glass tissue grinder (Wheaton Science Products). The RNeasy Mini Kit (Qiagen) was used for RNA isolation according to the manufacturer's protocol. The RNA yield and the ratio of absorbance at 260 nm to 280 nm (A_260_/A_280 _ratio) and 260 nm to 230 nm (A_260_/A_280 _ratio) were measured with the NanoDrop^® ^ND-1000 Spectrophotometer (NanoDrop Technologies). The integrity of isolated total RNA was assessed with the RNA 6000 Nano LabChip^® ^kit using the Agilent 2100 Bioanalyzer (Agilent Technologies). The Agilent 2100 Expert software was used to generate an integrity measure for RNA called the RNA Integrity Number (RIN) [27] as a criterion of RNA quality for downstream experiments. The RIN value range from 10 (intact) to 1 (totally degraded) [27,48].

The precipitated RNA was resuspended in 50 μl DEPC water and 0.5 μg of RNA was reverse transcribed with 200 U Superscript™ III Reverse Transcriptase (Invitrogen) for 60 min at 50°C using Oligo-dT in 20 μl volume. All reverse transcription reactions were carried out in parallel to avoid introducing experimental variation.

### Selection of candidate housekeeping genes

A selection of 7 housekeeping genes (*ACTB*, *GAPDH*, *HPRT*, *YWHAZ*, *ARBP*, *SDHA *and *UBC*) belonging to different functional classes were selected to reduce the chance that these genes might be co-regulated (Table 1). These were pre-designed, gene-specific TaqMan^® ^primer and probe sets (TaqMan^® ^Gene Expression Assays, Applied Biosystems) (Table 2).

### Real-time quantitative RT-PCR

PCR was performed using the ABI Prism^® ^7500 Real-Time PCR System (Applied Biosystems) in 96 well microtitre plates using a final volume of 10 μl. Amplifications were performed starting with a 2 min activation step for AmparaseUNG at 50°C, 10 min template denaturation step at 95°C, followed by 40 cycles of 95°C for 15 s and 60°C for 1 min.

### Analysis of expression stability

The Q-RT-PCR raw data was analysed using the SDS 7500 software, (Applied Biosystems). The Q-RT-PCR data was converted to linear values compatible with the geNorm and NormFinder programs. For stability comparisons of candidate housekeeping genes within sample groups, the software geNorm, version 3.4 [29] (Visual Basic application tool for Microsoft Excel) and NormFinder [30] (a Microsoft Excel Add-in) were used according to developer's recommendations. For comparisons between groups (relative to control), stability was determined by both normalising to total RNA, and via normalising to the geometric mean of multiple housekeeping genes [29,35]. In addition, the option in NormFinder to define groups was applied to the different gland states. The software program, SPSS version 15 was used to carry out statistical analysis, namely the Mann-Whitney-U Test (statistical significance <0.05), of gene expression among experimental groups.

Gene expression analysis of *SMGB *in the 3 groups (normal, atrophic and regenerative) was carried out using the relative expression software tool (REST^©^) [31], developed for group-wise comparison and statistical analysis of relative expression results and includes an efficiency correction for real-time PCR efficiency of the individual transcripts.

### Statistical analysis

Gland weight results were expressed as the mean ± SEM (Standard Error of the Mean), and were statistically compared by paired Student's t-test. P < 0.05 was considered statistically significant. The software program, SPSS version 15 was used for further statistical analysis of gene expression using the Mann-Whitney-U Test (statistical significance <0.05). The REST^© ^statistical model uses a pairwise fixed reallocation randomisation test (statistical significance <0.05).

## Abbreviations

*ACTB*: β-actin; *ARBP*: acidic ribosomal phosphoprotein P0; cDNA: complementary DNA; Ct: cycle threshold; *GAPDH*: glyceraldehyde 3-phosphate dehydrogenase; *HPRT*: hypoxanthine-guanine phosphoribosyltransferase; mRNA: messenger RNA; NTC: no template control; PCR: polymerase chain reaction; Q-RT-PCR: quantitative real-time PCR; RIN: RNA integrity number; *SDHA*: succinate dehydrogenase complex, subunit A, flavoprotein; *SMGB*: submandibular gland protein B; StdDev: standard deviation; *UBC*: Ubiquitin C and *YWHAZ*: tyrosine 3-monooxygenase/tryptophan 5-monooxygenase activation protein, zeta polypeptide.

## Authors' contributions

NS performed all the experimental procedures and was the primary author of the manuscript. GHC participated in the study design and structuring of the manuscript. EC supplied tissue from 2 wk ligated followed by 3 day de-ligated submandibular glands. SO helped perform the surgical procedures. KLP aided in histological preparation of samples, while GP aided in structuring and editing of the paper. All authors read and approved the manuscript.
